# Emphysema prevalence related air pollution caused by a cement plant

**DOI:** 10.1186/s40557-016-0101-8

**Published:** 2016-04-07

**Authors:** Hyun Seung Lee, Chul Gab Lee, Dong Hun Kim, Han Soo Song, Min Soo Jung, Jae Yoon Kim, Choong Hee Park, Seung Chul Ahn, Seung Do Yu

**Affiliations:** Department of Occupational & Environmental Medicine, School of Medicine, Chosun University, 558 Pilmun-daero, Dong-gu Gwangju, 61453 Korea; Department of Radioloy, School of Medicine, Chosun University, 558 Pilmun-daero, Dong-gu Gwangju, 61453 Korea; National Institute of Environmental Research, 42 Hwangyong-ro, Seogu Incheon, 22689 Korea

**Keywords:** Emphysema, Cement, Air pollution, HRCT

## Abstract

**Background:**

To identify adverse pulmonary health effects due to air pollution derived from a cement plant in Korea. The emphysema prevalence in residents around a cement plant was compared to that in the group who live far away from the plant by chest films (PA and lateral view) and high-resolution computed tomography (HRCT) lung images.

**Methods:**

From June to August in 2013 and from August to November in 2014, chest films and HRCT scan were conducted on residents over the age of 40 who lived around a cement plant. The residents were divided into two groups; a “more exposed group (MEG)” which consisted of 1,046 people who lived within a 1 km radius and a “less exposed group (LEG)” which consisted of 317 people who lived more than 5 km away from the same plant. We compared the emphysema prevalence and estimated the OR of this between the MEG and the LEG by using a chi-square and logistic regression on chest films and HRCT.

**Results:**

The emphysema prevalence was 9.1 % in the LEG, 14.3 % in the MEG on chest films and 11.4 %, 17.8 % on the HRCT, respectively. The OR of the emphysema prevalence in MEG was 2.92 (95 % CI 1.77-4.83) on the chest films, 2.56 (95 % CI 1.64–3.99) on the HRCT after sex, age, body mass index (BMI), smoking history, residency period and firewood used history were adjusted. The OR in the less than 29 pack-years smoking history was 1.66 (95 % CI 0.92–3.06) and in the more than 30 pack-years was 3.05 (95 % CI 1.68–5.52) on the chest films, and was 1.68 (95 % CI 0.98–2.90), 2.93 (95 % CI 1.72–4.98) on the HRCT, respectively.

**Conclusion:**

The emphysema prevalence seems to be affected by the level of exposure to air pollution derived from the cement plant as well as sex, age, BMI, and smoking history in this study. Moreover, the OR of the case of the more exposed to the air pollution was similar to that of the case in smoking.

## Background

Exposure to air pollution has been associated with increases in morbidity and mortality due to respiratory and cardiovascular disease. Epidemiological and toxicological research results continued to support the association between air pollution and an increase in the incidence of those diseases [1–3]. Chronic obstructive pulmonary disease (COPD) among respiratory disease is characterized by persistent airflow limitation that is caused by a mixture of small airways disease (obstructive bronchiolitis) and parenchymal destruction (emphysema) [4]. Although cigarette smoking is the most powerful risk factor for COPD, other factors such as occupational exposures, environmental air pollution, genetic factors, lung development abnormalities, accelerated aging, bronchial hyper-reactivity, and socioeconomic statuses are associated with development and progression of COPD [4, 5]. Even if the epidemiological scientific evidence like a longer follow-up period of larger studies on the causal relationship between air pollution and COPD development is still not enough [6], air pollution generally exacerbates respiratory and cardiovascular disease [1, 7, 8], and high level of particulate is associated with an increase of COPD incidence and prevalence [9].

Owing to the air pollution around cement plants, complaints of residents who have lived near the cement plants were a social problem since the late 1970s in Korea. They have always complained of coughing, phlegm and shortness of breath, and thought that this phenomenon might be related to the dust coming from the plants. The emissions in the cement manufacturing process create the combustion such as particulates, carbon dioxide (CO_2_), nitrogen oxides, sulfur oxides, and heavy metals, and there is an additional cloud of dust generated from the mining and transportation [10–13]. Accordingly, the epidemiologic surveys have been conducted to assess the residents’ health effects of air pollution resulting from the cement plants by the Ministry of Environment (National Institute of Environmental Research) [14]. We reported the health effects of exposure to particulate matters around a cement plant on lung function of residents, which is one part of the epidemiologic survey results. The result was that ventilation impairment rate of pulmonary function test (PFT) is higher in the “more exposed group (MEG)” who lived within a 1 km radius of a cement plant than “less exposed group (LEG)” who lived more than 5 km away from the same plant [10].

However, there is a need for a more objective assessment to health effects of air pollution exposure because PFT results may vary depending on the efforts of examinees. The radiographic test such as chest films (PA and lateral view) and HRCT can be more objective evidence than PFT in that the former does not need the examinee’s efforts in the process of epidemiological survey. Emphysema, the term commonly used for COPD phenotype is defined as anatomically abnormal enlargement of the air spaces owing to destruction and deformation of alveolar walls. It’s not difficult to identify emphysema through chest films and HRCT. The radiographic features of emphysema are the increased lung volume, reduced vascularity, or both on chest films. On HRCT, emphysema is characterized by the presence of areas of abnormally low attenuation, which can be easily contrasted with surrounding normal lung parenchyma [15]. We compared the prevalence of emphysema through chest films and HRCT following the previous report about ventilation impairment between the ‘MEG (more exposed group)’ and ‘LEG (less exposed group)’ of residents living around the cement plants or limestone mines [10]. The objective of this study is to figure out whether there is a difference in emphysema prevalence according to the level of exposure to air pollution derived from a cement plant.

## Method

### Participants

The epidemiological surveyed cement plant is located in a rural town near a big city, and about 13,000 residents live in this town. The survey was done twice with the primary survey conducted from June to September 2013 and additional investigations from August and November 2014. The residents who lived within the 1 km radius of the cement plant were designated as the MEG (case group), the LEG (control group) lived more than 5 km away from the cement plant in an area. Two groups were similar in socioeconomic living conditions of a rural region except for the direction of the wind centering on the plant [10]. Since the origins of COPD can occur from childhood or even in utero [5], it is important to consider the degree of air pollution from the past to the present. However, there is no air pollution data from the past, only being one data measured as a total of 21 days in June, August and October 2103 at the time of the epidemiological survey in the areas surrounding the cement plant [10]. Sufficient explanation about the purpose of the epidemiologic survey was given to residents living around the cement plant area prior to the research [10]. In addition, many of them volunteered to participate in it. MEG (2,287 residents estimated to live there) and LEG (estimated 580 residents) are over 40 years old (total 2,867), and if they were engaged in the cement plant-related working in the past, they were excluded in this analysis (134 among chest x-ray was taken 1,497). The result was as follows: The analyzed participants in the MEG and LEG were 1,046 (45.7 % of 2,287) and 317 (54.6 % of 580) respectively, and a total number was 1,363 (47.5 % of 2,867). Written informed consent was provided before participation. Ethical approval for the study was approved by Institutional Review Board (IRB) of National Institute of Environmental Research.

### Radiographic data

We took chest films, chest PA and lateral view of the entire participants as a screening test, and HRCT was taken to confirm the people suspicious of emphysema among them. We used a direct digital radiographic (DDR) x-ray systems with automatic exposure control (Titan-2000, Comed Medical Systems Co, Korea) in order to obtain chest films. HRCT images were taken according to a routine protocol of the Chosun university hospital that is the 3-mm thick axial scan during suspended full inspiration in supine position, and the 5-mm thick section of coronal and sagittal reconstruction images using the Aquilion ONE 640 (Toshiba, Japan). Both were taken at an interval of at least one week in 2013 survey, and the primary and second survey were done at an interval of about one year. The candidates who required HRCT scans were those with emphysema on the chest films, and with ventilation impairment on bronchodilator PFT at the primary survey done in 2013. In the second survey of 2014, HRCT scans were conducted for the candidates who could not take that test because of personal reasons in the primary survey. As a result, 376 (27.6 %) out of 1,363 participants took part in the HRCT, 48 (15.1 %) out of 317 in LEG and 328 (31.4 %) in MEG respectively. Radiographic data of both groups were randomly taken without a specific order at a convenient time for participants to visit the hospital.

In order to maintain the consistency and exclude inter-observer bias among radiologists, 18 years of experience pulmonary radiologist read the chest films and HRCT. In addition, to exclude the intra-observer bias of the pulmonary radiologist, the chest films and HRCT were blinded by mixing participants’ films with those of patients on a radiographic reading protocol about HRCT within the hospital. Reading bias is supposed to be relatively small because the HRCT reading in this study that lasted for 2 years from 2013 to 2014 was conducted in the conditions in which respiratory patients are mixed with other general patients in natural way. The emphysema was defined as lung voxels below −950 Hounsfield units in PACS (picture archiving and communication system). Among the various findings from the chest films and HRCT radiologic reading papers, we only chose and analyzed the indisputable case such as emphysema that is obvious to experts in pulmonary radiology [16–19]. There has already existed data about the obvious relationship between air pollution and its effects on lungs and even though there may be a little controversy when it comes to radiographic reading, the possibility of its affecting the results of this study is significant.

### Data analysis

We compared the prevalence of emphysema between LEG and MEG on the chest films and HRCT. Therefore, we evaluated the factors that can affect the occurrence of emphysema such as sex, age, body mass index (BMI), smoking, firewood use history, residency period and income status. The subjects were divided into three groups in BMI; more than 25 kg/m^2^ (obese), 20.0–24.9 kg/m^2^ and less than 19.9 kg/m^2^ (thin). In smoking history, we also divided this group into three; never, less than and more than 30 pack years. The residency period was divided into less than and more than 25 years living since the dust collection facilities of the cement plant had been incorporated in the mid-1980s [10]. Income status was divided into less than and more than one million won a month which is average income of the residents in the area. Then we calculated proportions of emphysema on chest films and HRCT respectively in the MEG and LEG based on sex, age, smoking and firewood use history, residency period, and income status. For the proportion of emphysema on HRCT images, the total number of each group was put in the denominator. We tested to identify whether a difference in emphysema prevalence between each of the factors using a chi-square test. We used logistic regression to estimate the odds ratio (OR) of emphysema prevalence on chest films and HRCT images, regarding emphysema presents as dependent variable, groups differently exposed to air pollution derived from the cement plant and other factors (sex, age, smoking and firewood use history, residency period, and income status) as independent variables. A *p*-value of less than 0.05 was considered statistically significant. All statistical analyses were performed in SPSS 21.

## Results

### General characteristics of the participants

Among the 317 in the LEG, 61.2 % were female and 38.8 % were male (Table 1). In the MEG 1,046 participants were included, 62.6 % female and 37.4 % male. The age distribution was 42.3 % over 70 years in the LEG and 40.5 % in the MEG. The residents whose BMI is over 25 kg/m^2^ were 42.9 % and less than 19.9 kg/m^2^ were 6.9 % in MEG, they were significant differences compared to those in LEG. The residency periods of two groups were similar. In addition, in the smoking history, LEG and MEG were very similar. However, in the firewood use history, 64.0 % in LEG were significantly higher than the 45.5 % in the MEG. In other words, LEG and MEG were similar groups in the general characteristics.

**Table 1:** Descriptive characteristics of study participants

Variables		LEG (*n* = 317)	MEG (*n* = 1,046)	Total (*n* = 1,363)	*P*-value
Sex	Female	194 (61.2)	655 (62.6)	849 (62.3)	0.684
	Male	123 (38.8)	391 (37.4)	514 (37.7)	
Age	≤59	98 (30.9)	297 (28.4)	395 (29.0)	0.337
	60–69	85 (26.8)	325 (31.1)	410 (30.1)	
	≥70	134 (42.3)	424 (40.5)	558 (40.9)	
BMI	≥25.0	113 (35.6)	449 (42.9)	562 (41.2)	0.002
	24.9–20.0	164 (51.7)	525 (50.2)	689 (50.6)	
	≤19.9	40 (12.6)	72 (6.9)	112 (8.2)	
Residency period	≤24	93 (29.3)	296 (28.3)	389 (28.5)	0.720
	≥25	224 (70.7)	750 (71.7)	974 (71.5)	
Smoking	Never	213 (67.2)	716 (68.5)	929 (68.2)	0.854
	≤29	51 (16.1)	155 (14.8)	206 (15.1)	
	≥30	53 (16.7)	175 (16.7)	228 (16.7)	
Firewood	Not use	114 (36.0)	570 (54.5)	684 (50.2)	0.000
	Use	203 (64.0)	497 (45.5)	679 (49.8)	
Income	≥100	107 (33.8)	330 (31.5)	437 (32.1)	0.461
	≤99	210 (66.2)	716 (68.5)	926 (67.9)	

*P*-value by chi-square test

MEG: ‘more exposed group’ who lived within the 1 km radius of the cement plant

LEG: ‘less exposed group’ who lived 5 km or more away from the cement plant in not related to usually wind direction

Age and Residency period: years, Body mass index (BMI); kg/m^2^, Smoking: pack-years, Income: ten thousand won (Korea)/month

### Emphysema prevalence and OR

On chest films, the emphysema prevalence was 14.3 % in the MEG, which was significantly higher than 9.1 % in the LEG (Table 2). The 21.4 % of emphysema prevalence in the male was significantly higher than 8.1 % in the female. As age increased, the emphysema prevalence got higher, 5.1 % in less 59 years of age, 11.2 % in 60–69, and 20.3 % in over 70 years of age. The lower BMI was, the higher the chance of developing emphysema was. In smoking history, emphysema prevalence in the non-smoker was 8.7 %, and in contrast, less than 29 pack-years smokers were 16.0 % and more than 30 pack-years was 28.5 %, which was significantly higher in the more smoking pack-years. Similarly, the emphysema prevalence was higher in the group with firewood using history. The prevalence of emphysema in HRCT images was generally higher than that in the chest films. The emphysema prevalence on HRCT images was 17.8 % in the MEG, which was significantly higher than 11.4 % in the LEG. The difference in emphysema prevalence between the two groups widened slightly more than in chest films. The same went for the difference in sex, aging, and smoking history.

**Table 2:** Prevalence of emphysema on chest films and HRCT

Variables		Number	Chest films		HRCT	
			N (%)	*p*-value	N (%)	*p*-value
Group	LEG	317	29 (9.1)	0.017	36 (11.4)	0.007
	MEG	1,046	150 (14.3)		186 (17.8)	
Sex	Female	849	69 (8.1)	0.000	84 (9.9)	0.000
	Male	514	110 (21.4)		138 (26.8)	
Age	≤59	395	20 (5.1)	0.000	24 (6.1)	0.000
	60–69	410	46 (11.2)		59 (14.4)	
	≥70	558	113 (20.3)		139 (24.9)	
BMI	≥25.0	562	32 (5.7)	0.000	50 (8.9)	0.000
	24.9–20.0	689	96 (13.9)		125 (18.1)	
	≤19.9	112	51 (45.5)		47 (42.0)	
Residency period	≤24	389	24 (6.2)	0.000	44 (11.3)	0.002
	≥25	974	155 (15.9)		178 (18.3)	
Smoking	Never	929	81 (8.7)	0.000	99 (10.7)	0.000
	≤29	206	33 (16.0)		42 (20.4)	
	≥30	228	65 (28.5)		81 (35.5)	
Firewood	Not use	684	71 (10.4)	0.003	94 (13.7)	0.011
	Use	679	108 (15.9)		128 (18.9)	
Income	≥100	437	33 (7.6)	0.000	48 (11.0)	0.000
	≤99	926	146 (15.8)		174 (18.8)	

*P*-value by chi-square test

MEG: ‘more exposed group’ who lived within the 1 km radius of the cement plant

LEG: ‘less exposed group’ who lived 5 km or more away from the cement plant in not related to usually wind direction

Age and Residency period: years, Body mass index(BMI); kg/m^2^, Smoking; pack-years, Income: ten thousand won (Korean)/month

Chest films; Chest PA and Lateral view, HRCT; High-resolution computed tomography

The OR values to each variable of chest films and HRCT images were adjusted for all variables through logistic regression (Table 3). On the chest films, the OR of the emphysema prevalence in the MEG was 2.92 times (95 % CI 1.77–4.83) higher than that in the LEG. The OR in male was 2.38 (95 % CI 1.37–4.13) higher than that of female. The OR in an increase in age, in 60–69 and over 70 years was 1.81 (95 % CI 0.96–3.40), 3.36 (95 % CI 1.79–6.31) higher than that in less than 59 years respectively. The OR in the degree of BMI, in 24.9–20.0 kg/m^2^ group and in less than 19.9 kg/m^2^ group was 2.83 (95 % CI 1.82–4.40), 16.38 (95 % CI 9.11–29.45) higher than that in residents whose BMI was over 25 kg/m^2^. The OR in the less than 29 pack-years smoking history was 1.66 (95 % CI 0.92–3.06) and 30 pack-years was 3.05 (95 % CI 1.68–5.52) higher than that in the non-smokers.

**Table 3:** Odds ratio of emphysema prevalence on chest films and HRCT

		Chest films		HRCT	
Variables		Unadjusted	Adjusted	Unadjusted	Adjusted
		OR (95 % CI)	OR (95 % CI)	OR (95 % CI)	OR (95 % CI)
Group	LEG	1			
	MEG	1.66 (1.09-2.53)	2.92 (1.77-4.83)	1.69 (1.15-2.47)	2.56 (1.64-3.99)
Sex	Female	1			
	Male	3.08 (2.23-4.26)	2.38 (1.37-4.13)	3.34 (2.48-4.50)	2.49 (1.53-4.07)
Age	≤59	1			
	60–69	2.37 (1.38-4.08)	1.81 (0.96-3.40)	2.60 (1.58-4.27)	2.61 (1.47-4.62)
	≥70	4.76 (2.90-7.81)	3.36 (1.79-6.31)	5.13 (3.25-8.09)	5.43 (3.04-9.69)
BMI	≥25.0	1			
	24.9–20.0	2.68 (1.76-4.07)	2.83 (1.82-4.40)	2.27 (1.60-3.21)	2.34 (1.61-3.39)
	≤19.9	13.84 (8.27-23.12)	16.38 (9.11-29.45)	7.40 (4.60-11.9)	8.08 (4.70-13.88)
Residency period	≤24	1			
	≥25	2.88 (1.84-4.50)	2.50 (1.50-4.18)	1.75 (1.23-2.50)	1.32 (0.87-1.99)
Smoking	Never	1			
	≤29	2.00 (1.29-3.09)	1.66 (0.92-3.06)	2.15 (1.44-3.20)	1.68 (0.98-2.90)
	≥30	4.18 (2.89-6.03)	3.05 (1.68-5.52)	4.62 (3.28-6.50)	2.93 (1.72-4.98)
Firewood	Not use	1			
	Use	1.63 (1.19-2.25)	1.26 (0.82-1.93)	1.46 (1.09-1.95)	1.24 (0.85-1.82)
Income	≥100	1			
	≤99	2.29 (1.54-3.41)	1.41 (0.84-2.36)	1.88 (1.33-2.64)	1.03 (0.65-1.62)

Logistic regression with adjusted for all variables, CI: confidence interval

MEG: ‘more exposed group’ who lived within the 1 km radius of the cement plant

LEG: ‘less exposed group’ who lived 5 km or more away from the cement plant in not related to usually wind direction

Age and Residency period: years, Body mass index (BMI); kg/m^2^, Smoking: pack-years, Income: ten thousand won (Korea)/month

Chest films; Chest PA and Lateral view, HRCT; High-resolution computed tomography

On the HRCT images, the OR of the emphysema prevalence in the MEG was 2.56 (95 % CI 1.64–3.99) higher than that in the LEG. The OR in male was 2.49 (95 % CI 1.53–4.07) higher than that of female. The OR in an increase in age, in 60–69 and over 70 years was 2.61 (95 % CI 1.47–4.62), 5.43 (95 % CI 3.04–9.69) higher than that in less than 59 years respectively. The OR in the degree of BMI, in 24.9–20.0 kg/m^2^ group and in less than 19.9 kg/m^2^ group was 2.34 (95 % CI 1.61–3.39), 8.08 (95 % CI 4.70–13.88) higher than that in residents whose BMI was over 25 kg/m^2^. The OR in the less than 29 pack-years smoking history was 1.68 (95 % CI 0.98–2.90) and 30 pack-years was 2.93 (95 % CI 1.72–4.98) higher than that of the non-smokers. The emphysema prevalence also was affected by sex, age, BMI, and smoking history, but also the level of exposure to dust from the cement plant had an impact on developing emphysema. Moreover, the OR of the case of the more exposed to the dust was similar to that of the case in smoking.

## Discussion

COPD and emphysema are the major leading cause of death in the World. COPD is defined as physiologically by persistent airflow limitation, while pulmonary emphysema is defined anatomically by destruction of interalveolar septae and loss of lung tissue. Emphysema is defined as pathological “condition of the lung characterized by abnormal, permanent enlargement of the air spaces distal to the terminal bronchiole, accompanied by destruction of alveolar walls” [20]. Emphysema overlaps only partially with COPD. Clinically COPD is an overlapping disease such as emphysema, chronic bronchitis, and small airways disease [4]. Normal physiological aging results in enlarged alveolar spaces and loss of lung elasticity, whereas in COPD there is destruction of the alveolar walls and fibrosis of peripheral airways [21]. Exposures to particulate matter in air pollution may contribute to the development of emphysema [22]. We were interested in whether there was more occurrence in emphysema when residents had been exposed to PM generated from a cement plant. Unlike PFT on the health effects of dust from the cement plant, taking the radiographic images doesn’t require the examinees’ efforts. Identifying emphysema on the chest films or HRCT is not so difficult to the skilled pulmonary radiologist, and there is not much debate about the reading results [16–19]. Therefore, if there is a difference in the emphysema prevalence in accordance to the degree of exposure to air pollution derived from the cement plant, it can figure out objectively whether it is true or not that the residents feel uncomfortable because of air pollutants that have been leaking continuously for more than 40 years. Therefore, we have attempted such an analysis.

In this cross-sectional study, emphysema prevalence was affected by sex, age, BMI, smoking history and the level of exposure to dust from the cement plant. Generally, emphysema is more pronounced in men than in women [23–29]. The possible explanation for the fact that emphysema is more common in men was that male smoking prevalence are two to three times that of females and males are more exposed to occupational harmful agents [26]. Another possible explanation is that while females have thicker small airway walls relative to luminal perimeters [24], in males the particles can penetrate deeper into the alveolar surface due to a larger airway caliber [26]. Sex hormones also seem to affect sex differences of COPD prevalence. Estrogen act as complex influences on lung growth and development, airway hyper responsiveness, detoxification of tobacco smoke, production of cytokines and triggering a TH2 dominant immune response [28]. BMI is inversely associated with emphysema prevalence [30–34]. But it is independent of age, sex, and smoking history [34]. Although the relationship between BMI and emphysema seems to be related to basal metabolic rate or nutritional depletion, it is unclear if emphysema predisposes to weight loss among COPD patients or whether low body weight contributes to the development of emphysema [32]. Therefore, the causal relationship between obesity and emphysema cannot be established [34]. Smoking is the most common risk factor for COPD [9]. In a systematic review with meta-analysis, the adjusted relative risk estimate of emphysema was 4.51 (95 % CI 3.38–6.02) for never smoker [35].

In this study, the OR of the emphysema prevalence in MEG was 2.92 after adjusted sex, age, BMI, smoking history, residency period and firewood used history. Moreover, the OR of MEG to air pollution derived from the cement plant was similar to in the more than 30 pack-years smoking history. Higher long-term air pollution exposures are associated with emphysema [22]. The mechanisms of emphysema development caused by smoking and air pollution are similar. Alveolar macrophages exposed to dust cause ROS generation, apoptotic cells induction and decreased glutathione, and secrete Interleukin-1β and TNF-α. In other words, the pulmonary toxicity is induced by endocytosis, oxidative stress induced-apoptosis and induction of pro-inflammatory cytokines when alveolar macrophages are exposed to dust [36]. Various air pollutants are discharged in the cement manufacturing processes, mining and transportation of limestone [10–13]. The carbon dioxide, nitrogen oxides (NOx), sulfur oxides (SO_2_) and aromatic hydrocarbons in the pyroprocessing to make clinker as well as calcium oxide (CaO), silica (SiO_2_), aluminum trioxide (Al_2_O_3_) iron oxide (Fe_2_O_3_), magnesium oxide (MgO) and other heavy metals contained in the cement products are discharged into the ambient air [12]. In a study of quantification of ROS and nitric oxide (NO) levels in serum of the residents who exposed to cement emission such as PM, NOx and SO_2_ [37], ROS was increased while NO was decreased. In another study [38], the phagocytic activity of polymorphonuclear neutrophils (PMNs) was significantly decreased among workers in a cement plant.

In this survey, to determine the concentration of particulate of air pollution in exposed residents, we collected particulate in the atmosphere using low volume sampler in a total of 21 days every seven days in June, August and October [10]. The mean PM_10_ concentration was 45.5 μg/m^3^ (95 % CI 37.8 ~ 53.3) in MEG living beside the cement plant, higher than 38.5 μg/m^3^ (95 % CI 32.3 ~ 44.7) in LEG living a 5 km away point from the cement plant. The mean PM_2.5_ concentration in MEG was 25.5 μg/m^3^ (95 % CI 18.7 ~ 32.3), higher than 19.3 μg/m^3^ (95 % CI 14.1 ~ 24.6) in LEG [10]. This concentration is less than annual mean of air quality guidelines defined by Korea Ministry of Environment (50 μg/m^3^ for PM_10_, 10 μg/m^3^ for PM_2.5_ μg/m^3^). However, the measured concentration in this study is close to the level at 24-h mean concentration of the WHO guidelines (50 μg/m^3^ for PM_10_, 25 μg/m^3^ for PM_2.5_ μg/m^3^). According to the data submitted by the cement plant during this survey, the concentration of total suspended particulates emitted from the plant was 7,856 tons/year in 1982, which is 258 times that of 2013. It can be assumed that residents who lived close to the cement plant were exposed to higher levels of particulate before dust collection facilities had been implemented in mid-1980s. Because many of the participants in this study are current farmers or people who farmed before, they were always exposed to dust in the soil and to chemical substances like agriculture chemicals [10]. Therefore, we do not claim that the residents were exposed only to the air pollution derived the cement plant. What we want to claim is that though the complex multiple factors such as sex, age, smoking, occupation and genetic factors, etc. contribute to emphysema development, air pollution resulting from the cement plant had an impact that. According to a systematic review and meta-analysis [9], the 10 μg/m^3^ increment of PM_10_ in quantitative effects of outdoor air pollution was associated with increase of 1 ~ 6 % in COPD mortality in the case of short-term exposure, while in the case of chronic exposure 10 % increase in mortality was shown. In the other data related to this survey, 70 % of the residents who were living in the area when the cement plant was first built have moved to larger cities now, and the standardized lung cancer incidence and mortality of the remaining residents due to respiratory diseases were slightly higher than those of the whole Korean population. There is also a possibility that the residents whose health was compromised have already died of emphysema or lung cancer [10].

A strength of this study was that the participants are community population, not hospital based subjects. The estimated residents who were more than 40 years old were 2,287 in MEG and 580 in LEG at this survey point, among whom the 1,497 (52.2 %) participated in the chest radiological test. These results in this study of 1,363 among 1,497 except for persons who were engaged in the cement plant-related working in the past occupational history were analyzed. In particular, more than 70 % of residents who were over 60-year-olds and had more than 40-year residence were analyzed. In addition, the MEG and LEG are similar people living in the same rural area with similar occupational (mostly farmers and a small number of merchants), economic and socio-cultural settings except the degree of the exposure to dust from the cement plant depending on how far away they live from the plant and the direction of the wind [10]. Therefore, even though this is a cross-sectional study with small population, it can be interpreted to reflect the result of the exposure to the cement particles for a long time.

There are some limitations on this study. HRCT scan of all the subjects was not taken because of survey cost and radiation risk. HRCT was done to residents who were suspected to have emphysema on the chest films and ventilation impairment on bronchodilator PFT. Therefore, there may be an argument over the fact that the whole participants were used as the denominator in calculating the prevalence of emphysema on HRCT. However, the presence of emphysema on chest films can be confirmed through HRCT, which is considered as a method of serial screening test. And, reading and interpretation done by the only one 18 years of experience pulmonary radiologist expert can be something debatable, and also a major weakness of this manuscript. But it is too basic procedure to university hospital pulmonary radiologists to interpret emphysema. Besides, if need be, the emphysema images with lung voxels below −950 Hounsfield units are easily evaluated by visual checking in PACS. Moreover, we didn’t take quantitative measurement of emphysema because there was no need in radiologic interpretation routine work and the purpose this research was to see the presence of emphysema, which can be another limitation.

## Conclusion

Despite some limitations of this study, such as cross-sectional study with small population, emphysema prevalence in more exposed group to air pollution derived from the cement plant was higher than that in the comparative group after all the factors affecting emphysema development such as sex, age, BMI, smoking were adjusted. It means that air pollution derived from the cement plant may have an impact on the health of residents living around the plant.
